# A Simple Extraction Method of Young’s Modulus for Multilayer Films in MEMS Applications

**DOI:** 10.3390/mi8070201

**Published:** 2017-06-23

**Authors:** Xin-Ge Guo, Zai-Fa Zhou, Chao Sun, Wei-Hua Li, Qing-An Huang

**Affiliations:** Key Laboratory of MEMS of the Ministry of Education, Southeast University, Nanjing 210096, China; guoxinge93@outlook.com (X.-G.G.); sun_chaos@seu.edu.cn (C.S.); liwh@seu.edu.cn (W.-H.L.); hqa@seu.edu.cn (Q.-A.H.)

**Keywords:** resonance frequency, cantilever, FEM, thin film, Young’s modulus

## Abstract

Based on the first resonance frequency measurement of multilayer beams, a simple extraction method has been developed to extract the Young’s modulus of individual layers. To verify this method, the double-layer cantilever, as a typical example, is analyzed to simplify the situation and finite element modeling (FEM) is used in consideration of the buckling and unbuckling situation of cantilevers. The first resonance frequencies, which are obtained by ANSYS (15.0, ANSYS Inc., Pittsburgh, PA, USA) with a group of thirteen setting values of Young’s modulus in the polysilicon layer are brought into the theoretical formulas to obtain a new group of Young’s modulus in the polysilicon layer. The reliability and feasibility of the theoretical method are confirmed, according to the slight differences between the setting values and the results of the theoretical model. In the experiment, a series of polysilicon-metal double-layer cantilevers were fabricated. Digital holographic microscopy (DHM) (Lyncée Tech, Lausanne, Switzerland) is used to distinguish the buckled from the unbuckled. A scanning laser Doppler vibrometer (LDV) (Polytech GmbH, Berlin, Germany) system is used to measure the first resonance frequencies of them. After applying the measurement results into the theoretical modulus, the average values of Young’s modulus in the polysilicon and gold layers are 151.78 GPa and 75.72 GPa, respectively. The extracted parameters are all within the rational ranges, compared with the available results.

## 1. Introduction

Material mechanical parameters, such as Young’s modulus, residual stress, and so on, not only have great effect on the functions of MEMS (Micro-Electro-Mechanical System) devices, but also have great influence on yield, service life, and the work reliability of MEMS devices. However, it is a challenging task to detect and measure the mechanical properties of thin films and membrane structures for MEMS applications [1,2,3,4]. In surface micromachining technology, with the same membrane structures, different processes will lead to different material properties. The different environment of a process, such as temperature and irradiation, will also cause different properties of the materials. Even from the same material, process, reactor, and environment when testing, there will also be differences among the material properties of thin films. Thus, the mechanical parameters need to be accurately measured in situ. As for the present measuring methods for mechanical parameters of thin film materials, most of them are suitable for single-layer thin film materials. For the micromachining technology of some materials, these methods for the single-layer thin film are no longer applicable. Therefore, an in situ extracting method of material properties [5,6] for multilayer films is expected. Until now, various theoretical methods have been presented for extracting mechanical parameters of multilayer films, such as the resonance frequency method [7] and pull-in voltages [3,8].

In this paper, a novel approach was proposed to extract the Young’s modulus of each layer with multilayer beams using resonance frequency measurement. Firstly, a model of the resonance frequency using multilayer beams with different widths was introduced. Then, taking the buckled and unbuckled situations of multilayer beams into consideration, a method was presented to extract the Young’s modulus in each layer by designing test structures. The presented theoretical model was verified by FEM methods. Finally, some test structures, as a typical example, were fabricated and a scanning laser Doppler vibrometer (LDV) (Polytech GmbH, Berlin, Germany) system was used to measure their first resonance frequencies. The results turned out to be in good agreement with the rational values in the available literature. Without applying residual stress into the model and calculations, the test approaches and the calculation procedures became simple and easy. 

## 2. Theory

### 2.1. Theory Model of the Multilayer Beam

**Figure 1:**
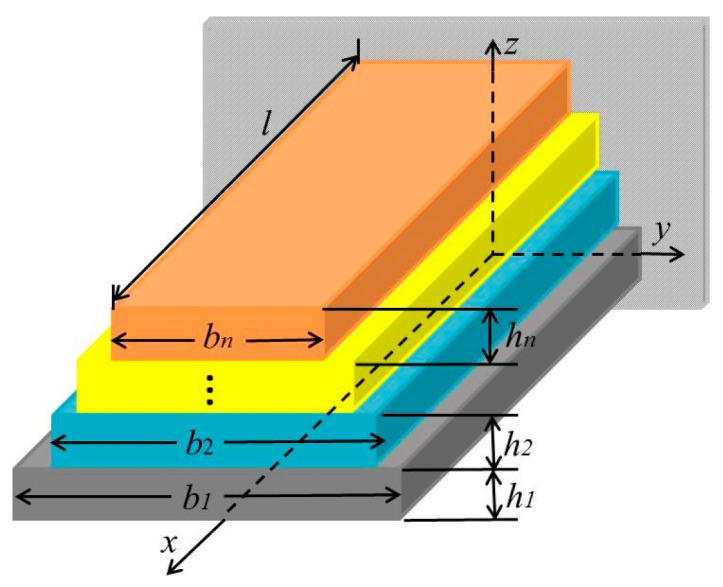
3D view of an n-layer beam with different widths.

The approach is to extract the Young’s modulus of each layer based on multilayer beams, and the cross-section of an n-layer beam with different widths is shown in Figure 1. The length, width, and height direction of the beam are along the *x*, *y*, and *z* axis, respectively. The length of the beam is *l*. The width, thickness, Young’s modulus, Poisson’s ratio, and density of the *i*th layer are *w_i_*, *h_i_*, *E_i_*, *ν_i_*, and *ρ_i_* respectively.

For composite multilayer beams studied in the paper, the widths are all five times longer than the thicknesses, and the stress-strain relation should be analyzed under the plain-strain condition. Thus, the Young’s modulus *E_i_* should be substituted by the effective Young’s modulus ${\tilde{E}}_{i}$, as shown in Equation (1):

(1) $${\tilde{E}}_{i}=\{\begin{array}{cc} E_{i} & ,\;w_{i}<5h_{i}\\ \frac{E_{i}}{1-{\nu_{i}}^{2}} & ,\;w_{i}\geq 5h_{i} \end{array}$$

As shown in Figure 1, the height of the bottom, *z*_0_, is 0 and the height of the *i*th layer is *z_i_*. Thus, the expression of *z_i_* is shown in Equation (2):

(2) $$z_{i}=\sum_{j=1}^{i}h_{i}$$

The distance of the neutral axis and the bottom of the beam is *z_c_*, which is expressed as [9]:

(3) $$z_{c}=\frac{\sum_{i=1}^{n}{\tilde{E}}_{i}w_{i}\left(z_{i}^{2}-z_{i-1}^{2}\right)}{2\sum_{i=1}^{n}{\tilde{E}}_{i}w_{i}\left(z_{i}-z_{i-1}\right)}$$

The moment of inertia of the *i*th layer, *I_i_*, with respect to the neutral axis of the beam is expressed in Equation (4):

(4) $$I_{i}=\int_{i}{\left(z-z_{c}\right)}^{2}dA_{i}=\frac{1}{3}w_{i}\left[{\left(z_{i}-z_{c}\right)}^{3}-{\left(z_{i-1}-z_{c}\right)}^{3}\right]$$

Here *A_i_* is the cross-section area of the *i*th layer. The bending stiffness $\bar{EI}$ and the linear density $\bar{\rho A}$ of the beam are shown below [10]:

(5) $$\bar{EI}=\sum_{i=1}^{n}{\tilde{E}}_{i}I_{i}$$

(6) $$\bar{\rho A}=\sum_{i=1}^{n}\rho_{i}A_{i}$$

### 2.2. The Deduction of the Curvature Radius

The paper takes multilayer cantilevers as the test structures. For a multilayer cantilever, if the compressive stress of the top layer is predominant, the cantilever will have an upward deflection. If the tensile stress of the top layer is predominant, the cantilever will have a downward deflection. Both the upward and downward deflection has the same effect on the modeling and results. However, if the downward deflection is too large to adhere to the substrate, cantilevers are difficult to, or cannot, be vibrated independently and the results from these structures are invalid and discarded. Therefore, the model proposed is suitable in small residual stress situations. Models, which can compensate the influence of the large residual stress in the thin films, are shown in [11,12,13].

**Figure 2:**
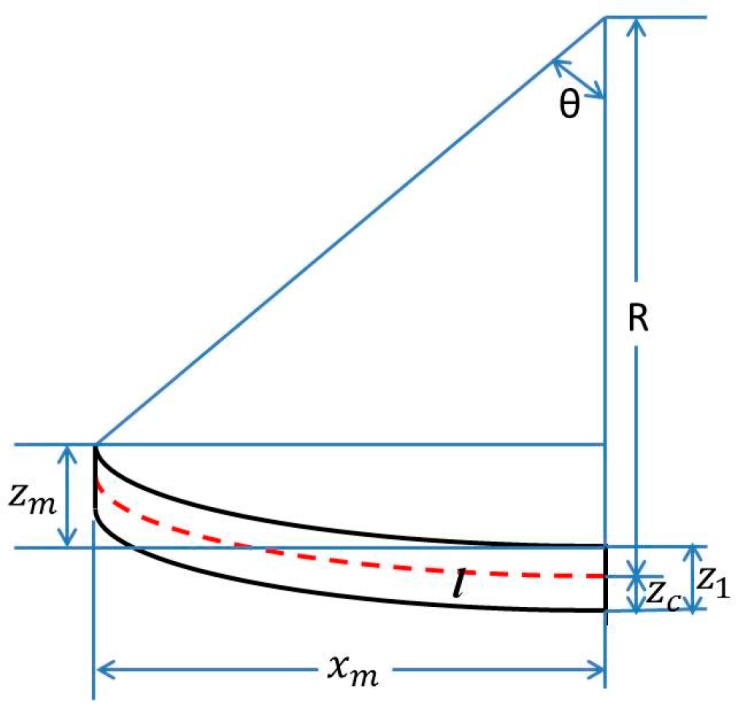
Schematic of a buckled multilayer beam.

The paper takes the upward deflection, for example, and assumes that the buckled multilayer cantilever has a uniform curvature radius, as shown in Figure 2. Figure 2 shows the relation between the curvature radius and the maximum deflection of the multilayer cantilever, as expressed by Equation (7):

(7) $$\{\begin{array}{c} \;\theta \;=\;l/R\;\\ \cos\left(\theta \right)=[R-(z_{1}-z_{c})-z_{m}]/[R-(z_{1}-z_{c})] \end{array}$$

In Figure 2, *R* is the curvature radius of the multilayer cantilever, *z_m_* is the maximum deflection of cantilever, *l* is the length of the unbuckled cantilever, *x_m_* is the length of buckled cantilever, *z*_1_ is the thickness of the multilayer cantilever and *z_c_* is the height of the neutral axis. By solving Equation (7), values of the curvature radius can be obtained accurately. Generally, the maximum deflection of the beam is much smaller than the length of the beam, so *x_m_* can be approximated to *l*. Furthermore, *z*_1_ − *z_c_* is also much smaller than the curvature radius of the cantilever, so after simplification and ignoring *z*_1_ − *z_c_*, z*_m_* can be expressed by $z_{m}=R-R\cos\theta$. Using the half-angle formula $2\sin^{2}\frac{\theta }{2}=1-\cos\theta$ and the approximately simplification $\sin\frac{\theta }{2}=\frac{l}{2R}$, a simplified equation, which is shown in Equation (8), can be obtained:

(8) $$R=l^{2}/(2z_{m})$$

### 2.3. The Theoretical Deduction for Material Parameters

If the longitudinal gradient residual stress is neglected, the single-layer cantilever will remain unbuckled after release. The strain resulting from the residual stress tends to have less effect on the vibration. Therefore, it is considered that the resonance frequency has nothing to do with the residual stress when the single-layer cantilever remains unbuckled after release [14]. However, due to the unmatched thermal stress, or other reasons, in most cases the multilayer cantilever will be buckled after release. The deflection has an effect on the resonance frequency of the cantilever after release [15,16].

For unbuckled multilayer cantilevers after release, the approximate analytic formula of its first resonant frequency is shown in Equation (9):

(9) $$f_{1ucf}=\frac{1.875^{2}}{2\pi l^{2}}\sqrt{\frac{\bar{EI}}{\bar{\rho A}}}$$

here subscript 1 represents the first resonant frequency, *u* represents that the cantilever is unbuckled, and *cf* represents that the boundary condition is cantilever.

When the cantilever is buckled, the influence of deflection to resonance frequency cannot be ignored. Assuming that *R* is the curvature radius of cantilever after release, a function describing its first resonant frequency is shown in Equation (10) [15,16,17]:

(10) $$\begin{array}{l} \frac{1}{\sqrt{\chi_{n}^{2}-1}}\sin\left(\sqrt{\chi_{n}+1}\frac{l}{R}\right)\sinh\left(\sqrt{\chi_{n}-1}\frac{l}{R}\right)+\\ \cos\left(\sqrt{\chi_{n}+1}\frac{l}{R}\right)\cosh\left(\sqrt{\chi_{n}-1}\frac{l}{R}\right)+1=0, \end{array}$$

where:

(11) $$\chi_{n}^{2}=\bar{\rho A}R^{4}{\left(2\pi f_{n}\right)}^{2}/\bar{EI}.$$

Equation (10) is a transcendental equation. Except rare cases (such as the simple trigonometric equation), this kind of equation can only be solved approximately using numerical methods. There are many approximate methods to solve the transcendental equation. Common methods are Newton’s method, dichotomy, Mueller’s method, and so on [18,19]. There are numerous sets of solutions $\chi_{n}$ for Equation (13). Each $\chi_{n}$ has the corresponding *i*th resonance frequency. However, to ensure that the first resonance frequency *f_1_* is obtained, the solution of Equation (10) should be the smallest one, $\chi_{1}$.

If the curvature radius of the cantilever tends to infinity, take the limit of Equation (10) and the result is the same as Equation (9), which means when the curvature radius of the cantilever tends to infinity, the vibrations of both the buckled and unbuckled multilayer cantilever have little difference. Thus, in this situation, Equation (9) can describe its resonance frequency approximately.

### 2.4. The Extraction of Material Parameters

Under buckled or unbuckled situations of *n*-layer cantilevers, it only needs *n* types of cantilevers to obtain the Young’s modulus of each layer. The widths of the *j*th cantilever are *w_j_*_1_, *w_j_*_2_, …, *w_jn_* and the widths of the *k*th cantilever are *w_k_*_1_, *w_k_*_2_, …, *w_kn_*. If they have the same length, the vector (*w_j_*_1_, *w_j_*_2_, …, *w_jn_*) and the vector (*w_k_*_1_, *w_k_*_2_, …,*w_kn_*) should be linearly independent [20]. DHM (Lyncée Tech, Lausanne, Switzerland) was used to distinguish the buckled from the unbuckled and determined using Equation (9) or Equation (11). By applying the smallest value $\chi_{1}$ into Equation (11), the changed form of Equation (11) is shown in Equation (12):

(12) $$f_{1bcf}=\frac{1}{2\pi }\cdot {\left(\frac{\chi_{1}^{2}\cdot \bar{EI}}{\bar{\rho A}\cdot R^{4}}\right)}^{1/2}$$

Here, *b* represents that the cantilever is buckled. Assuming that the first resonance frequencies measured are *f*_1_, *f_2_*,…, *f_n_*, a set of equations is deduced from the theoretical formulas, as shown in Equation (13):

(13) $$\left\{ \begin{array}{l} f_{1xcf,1}({\tilde{E}}_{1},{\tilde{E}}_{2},\ldots ,{\tilde{E}}_{n})-f_{1}=0\\ f_{1xcf,2}({\tilde{E}}_{1},{\tilde{E}}_{2},\ldots ,{\tilde{E}}_{n})-f_{2}=0\\ \ldots \\ f_{1xcf,n}({\tilde{E}}_{1},{\tilde{E}}_{2},\ldots ,{\tilde{E}}_{n})-f_{n}=0 \end{array}\right.,$$

where:

$$x=\left\{ \begin{array}{l} b,\;\mathrm{when}\;\mathrm{the}\;\mathrm{cantilaver}\;\mathrm{is}\;\mathrm{buckled}.\\ u,\;\mathrm{when}\;\mathrm{the}\;\mathrm{cantilaver}\;\mathrm{is}\;\mathrm{unbuckled}. \end{array}\right.$$

Since each equation in Equation (13) is the linear equation, it can be shown in matrix form, which is shown in Equation (14):

(14) $$A\left[\begin{array}{c} {\tilde{E}}_{1}\\ {\tilde{E}}_{2}\\ \cdots \\ {\tilde{E}}_{n} \end{array}\right]=\left[\begin{array}{c} {f_{1}}^{2}\\ {f_{2}}^{2}\\ \cdots \\ {f_{n}}^{2} \end{array}\right],$$

where:

$$A=\left[\begin{array}{cccc} a_{1}I_{11} & a_{1}I_{12} & \cdots & a_{1}I_{1n}\\ a_{2}I_{21} & a_{2}I_{22} & \cdots & a_{2}I_{2n}\\ \vdots & \vdots & \cdots & \vdots \\ a_{n}I_{n1} & a_{n}I_{n2} & \cdots & a_{n}I_{nn} \end{array}\right],a_{i}=\{\begin{array}{c} \frac{1.875^{4}}{4\pi^{2}{l_{i}}^{4}{\bar{\rho A}}_{i}}\;\mathrm{when}\;\mathrm{the}\;\mathrm{cantilever}\;\mathrm{is}\;\mathrm{unbuckled}.\\ \frac{1}{4\pi^{2}}\frac{\chi_{1i}^{2}}{{\bar{\rho A}}_{i}{R_{i}}^{4}}\;\mathrm{when}\;\mathrm{the}\;\mathrm{cantilever}\;\mathrm{is}\;\mathrm{buckled}. \end{array}$$

$I_{ij}$ represents the moment of inertia of *j*th layer of the *i*th cantilever. $l_{i}$, ${\bar{\rho A}}_{i}$, $\chi_{1i}^{2}$, and $R_{i}$ represent the length, the linear density, the smallest value $\chi_{1}$, and the curvature radius of the *i*th cantilever, respectively. Since vectors which consist of widths of all of the cantilevers are linearly independent, the matrix A is invertible. Therefore, Equation (13) is solvable. 

Effective Young’s modulus ${\tilde{E}}_{i}$ will be obtained from Equation (13) rather than Young’s modulus *E_i_* because the Poisson’s ratio *ν_i_* in each layer is unknown. To make results more intuitive, the paper sets exact values to the Poisson’s ratio in each layer. The way to obtain Poisson’ ratio *ν_i_* is shown in [21,22,23]. The Poisson’s ratio of each layer in a certain rational range has little effect on the results of the Young’s modulus. For example, the rational range of the Poisson’s ratio in the polysilicon layer is 0.2~0.25 and the paper assumes 0.22 as the Poisson’s ratio in the polysilicon layer. It is obvious that results obtained from this will have little differences among the results obtained from the Poisson’s ratio in the polysilicon layer is 0.2 or other values in this rational range.

Solving Equation (13) will give many sets of results, but only one of them is required. Thus, it is necessary to select only one rational result from them. For example, the Young’s modulus of polysilicon has been reported in the range of 120 GPa~201 GPa [20]. Thus, only one result in this range is correct.

## 3. Finite Element Modeling

The double-layer cantilever adopted in this paper can simplify the situation. To verify the validity of the theoretical model, a set of double-layer cantilevers was analyzed by the FEM method. Since ANSYS (15.0, ANSYS Inc., Pittsburgh, PA, USA) cannot express the residual stress after release, heating the structure in advance simulated the buckled cantilever. From bottom to top, the first layer of the cantilevers was designed to be a polysilicon material, and the second layer was designed to be metal material: gold. Assuming that the parameters of the metal layer are fixed, the only changes to the Young’s modulus are in the polysilicon layer.

An available range of Young’s modulus of polysilicon is from 120 GPa to 180 GPa. This paper takes thirteen values between it with the interval of 5 GPa and obtains thirteen values of the first resonance frequency and the maximum deflection of the cantilevers, respectively, for theoretical deduction. Dimensions and parameters of the cantilevers (except the Young’s modulus of the polysilicon layer) are shown in Table 1.

**Figure 3:**
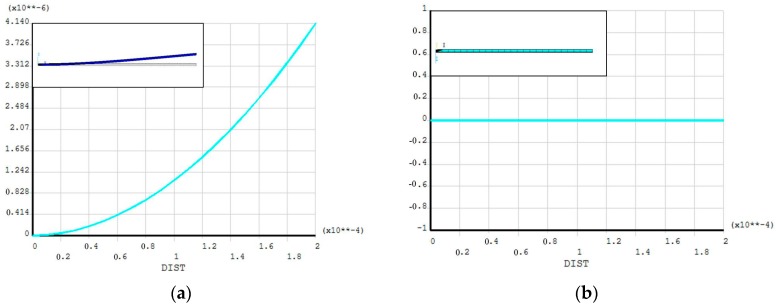
Pictures of the structure and deflection values of the cantilever. (**a**) The cantilever is buckled. (**b**) The cantilever is unbuckled.

Taking the Young’s modulus in the polysilicon layer as 120 GPa as an example, pictures of a polysilicon-metal double-layer cantilever structure and pictures of the deflection values of the cantilever (shown in Figure 3) can be obtained by using ANSYS (15.0, ANSYS Inc., Pittsburgh, PA, USA) in both unbuckled and buckled situations.

## 4. Simulation Results

When the difference between the curvature radius of the cantilever and the length of the cantilever is not obvious, the cantilever has a large deflection. In this case, through its curvature radius and the first resonance frequency, which is from ANSYS (15.0, ANSYS Inc., Pittsburgh, PA, USA), theoretical values of the Young’s modulus in the polysilicon layer can be deduced accurately by solving Equations (10) and (11).

When the cantilever has a small deflection, the deflection can be ignored approximately. In this case, the theoretical values of the Young’s modulus in the polysilicon layer can be obtained by using Equation (9) through its first resonance frequency of the cantilever. 

In both buckled and unbuckled situations of the cantilevers, the first resonance frequency is obtained by ANSYS (15.0, ANSYS Inc., Pittsburgh, PA, USA), the maximum deflection of the cantilevers, the curvature radius deduced, the theoretical values of the Young’s modulus in the polysilicon layer and the comparison between setting values of the Young’s modulus in the polysilicon layer, as well as ones in the theoretical model, are shown in Table 2 and Table 3.

From Table 2 and Table 3, the differences between the setting values of the Young’s modulus in the polysilicon layer and the values in the theoretical model are both not obvious. As the Young’s modulus in the polysilicon layer increases, the curvature radius of the cantilever increases. For relatively large curvature radii, errors from the calculations have less of an effect on its veracity. Thus final errors decrease as the Young’s modulus in the polysilicon layer increases. In addition, comparing the buckled situation with the unbuckled situation, slight differences between the theoretical values of the Young’s modulus in the polysilicon layer and errors in the unbuckled situation are all less than the ones in the buckled situation. This means that the cantilever does not have much deflection and using Equation (9), therefore, is more accurate. The reliability and feasibility of the theoretical model is demonstrated by slight errors which are all less than 8%.

Some of the reasons why setting values and theoretical values have differences are as follows. Firstly, conditions in the theoretical model and the actual ones are not exactly the same. Secondly, when simulating, there might exist some uncontrollable factors which lead to the simulation environment being different from the ideal environment. Thirdly, the deduction of the curvature radius of the cantilever will bring in inevitable errors.

## 5. Experiments and Discussion

Test structures, which are double-layer cantilevers for simplification, were fabricated using the MEMSCAP PolyMUMPS (MEMSCAP Inc., Durham, NC, USA) process based on surface micromachining technology. Before measuring the first resonance frequency with an LDV (Polytech GmbH, Berlin, Germany) system with a hardware modulus of MSV-400-M2, DHM (Lyncée Tech, Lausanne, Switzerland) (R2200) was used to determine whether cantilevers had deflection and to obtain the values of the curvature radius. Principles of LDV (Polytech GmbH, Berlin, Germany) and DHM (Lyncée Tech, Lausanne, Switzerland) are provided in the Supplementary Materials.

**Figure 4:**
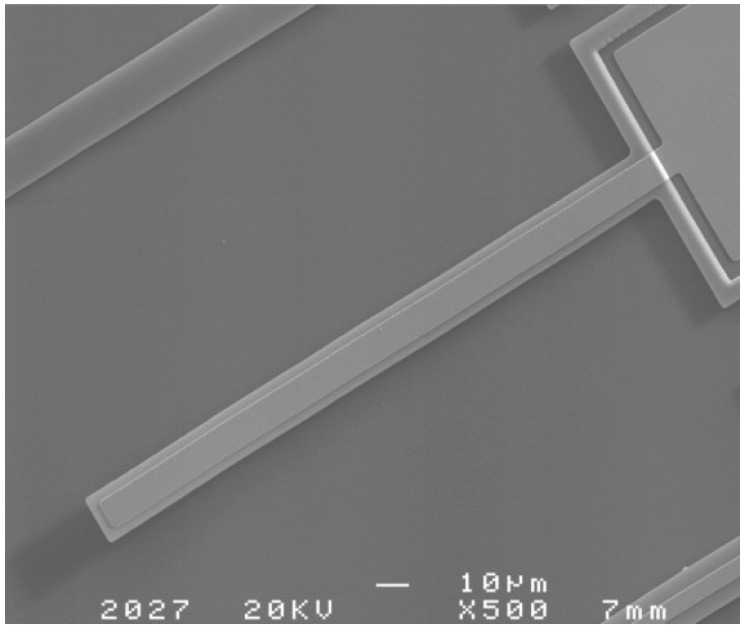
A SEM picture of cantilever 4.

For the double-layer cantilever, there are two unknown values of the Young’s modulus to obtain. Thus, two double-layer cantilevers with the same length but different widths should be grouped for calculation. The double-layer cantilevers were designed in two different lengths, and with the same length, widths of two double-layer cantilevers should be linearly independent. Cantilever 4, as an example, was shown in Figure 4 in scanning electron microscopy (SEM) photographs. The dimension, the deformation by DHM (Lyncée Tech, Lausanne, Switzerland), and the first resonance frequency measured of the cantilevers are shown in Table 4. To avoid the situation that the bottom layer of the cantilever adheres to the substrate, test structures with suitable dimensions should be designed. Simulated by ANSYS (15.0, ANSYS Inc., Pittsburgh, PA, USA), cantilever 1, whose residual stress is −20 MPa in the polysilicon layer and 20 MPa in the metal layer, have a downward deflection of −1.4304 μm. Residual stress of 20 MPa is enough to have a large deflection. Thus, compared to the thickness of the sacrificial layer of 2 μm, cantilevers, whose dimensions are little different from that of cantilever 1, may guarantee a relatively small possibility of substrate adhesion. However, it should be noted that the suitable dimension mentioned above is applicable to the MEMSCAP PolyMUMPS (MEMSCAP Inc., Durham, NC, USA) process and the dimensions of the test structures should be adjusted according to the specific process. In fact, a short time on release, which results when the multilayer cantilevers are relatively narrow, and the relatively large stiffness of multilayer cantilevers, leads to little possibility of adhesion.

Assuming that the cantilever has a uniform curvature radius *R*, curvature radii are obtained by numerical fitting from the deflection curves. The obtained curvature radii are shown in Table 5.

Assuming that the Poisson’ ratio and density of the polysilicon layer are 0.22 and 2330 kg/m^3^, respectively, and the Poisson’ ratio and density of the metal are 0.44 and 19,300 kg/m^3^, respectively, the Young’s modulus of each layer can be calculated by the first resonance frequencies of the test structures. Here is an example to use cantilever 1 and cantilever 2 to extract the Young’s modulus of each layer. After applying the material parameters and the first resonance frequencies in Equations (10) and (11), a system of two-element equations can be obtained, as shown in Equation (15):

(15) $$\left\{ \begin{array}{l} f_{1bcf,1}({\tilde{E}}_{1},{\tilde{E}}_{2})-f_{1}=0\\ f_{1bcf,2}({\tilde{E}}_{1},{\tilde{E}}_{2})-f_{2}=0 \end{array}\right.$$

Solve Equation (15) and find the only answer which meets the practical range of the material parameters. The reasonable answer is that the values of the Young’s modulus in the polysilicon layer and in the metal layer are 156.77 GPa and 68.54 GPa, respectively. Similarly, the Young’s modulus of each layer can also be extracted by cantilever 3 and cantilever 4. The results are shown in Table 6. From Table 6, it is obvious that the results agree with the practical range of material parameters and the values reported in [24].

There are some issues that affect the accuracy of the results. Firstly, in practical processes, photoetching, masking, and self-aligned processes can lead to dimension deviation between designed structures and processed ones. Generally, in surface micromachining technology, dimension deviations in a plane are no more than 0.5 μm and dimension deviations in thickness are no more than 0.02 μm. It will have an inevitable influence on minimized devices. Secondly, squeezed damping in air may lead to the inaccuracy of the first resonance frequency of the measured cantilevers [25,26]. Thus, the amplification factor for the frequency response functions at points of the resonance frequencies are needed to be greater than 20 [20].

## 6. Conclusions

In this paper, an approach of extracting the Young’s modulus of each layer for multilayer films was developed. Based on the first resonance frequency, a theoretical model was proposed for the multilayer beam. The multilayer cantilevers were adopted as the test structures, and buckled or unbuckled situations of the cantilevers were both considered. Its reliability and feasibility were confirmed theoretically by the FEM method with less than 8% error. In experiments, double-layer cantilevers have been fabricated for simplification. The Young’s modulus of each layer can be obtained by using two double-layer cantilevers with the same length, but different widths. The experimental results prove the accuracy of the presented approaches, and this study is suitable to extract the Young’s modulus of each layer for multilayer films.

## Figures and Tables

**Table 1:** Dimensions and parameters of the cantilevers.

Dimension/Parameter	Polysilicon Layer	Metal Layer
**Young’s modulus *E_i_* (GPa)**	--	57
**Residual stress** $\sigma_{i}$ **(MPa)**	10	−20
**Poisson ratio** $\nu_{i}$	0.22	0.35
**Density *ρ_i_* (kg/m^3^)**	2330	19,300
**Length *l* (μm)**	200	200
**Width *w_i_* (μm)**	30	30

**Table 2:** Results when the cantilever is buckled.

Setting Values of Young’s Modulus in Polysilicon Layer *E* (GPa)	The First Resonance Frequency from ANSYS (Hz)	The Maximum Deflection of Cantilever from ANSYS (μm)	The Curvature Radius (μm)	Theoretical Values of Young’s Modulus in the Polysilicon Layer *E*′ (GPa)	Error (%) $\left|(E-E^{\prime })/E\right|$
120	30,076	4.1403	4830.57	110.591	7.841
125	30,466	4.0334	4958.60	115.353	7.718
130	30,848	3.9237	5085.56	120.114	7.605
135	31,223	3.8376	5211.59	124.877	7.499
140	31,593	3.7476	5336.75	129.664	7.383
145	31,956	3.6622	5461.20	134.443	7.281
150	32,313	3.5810	5585.03	139.222	7.185
155	32,665	3.5038	5708.09	144.008	7.092
160	33,013	3.4301	5830.73	148.813	6.992
165	33,355	3.3598	5952.74	153.604	6.907
170	33,693	3.2926	6074.23	158.406	6.820
175	34,027	3.2283	6195.21	163.216	6.734
180	34,356	3.1667	6315.72	168.016	6.658

**Table 3:** Results when the cantilever is unbuckled.

Setting Values of Young’s Modulus in Polysilicon Layer *E* (GPa)	The First Resonance Frequency from ANSYS (Hz)	Theoretical Values of Young’s Modulus in the Polysilicon Layer *E*″(GPa)	Error (%) $\left|(E-E^{\prime \prime })/E\right|$
120	30,074	110.650	7.792
125	30,464	115.413	7.670
130	30,846	120.173	7.560
135	31,221	124.937	7.454
140	31,590	129.711	7.349
145	31,954	134.503	7.239
150	32,311	139.282	7.145
155	32,663	144.069	7.052
160	33,011	148.874	6.954
165	33,353	153.665	6.870
170	33,691	158.468	6.784
175	34,025	163.278	6.698
180	34,354	168.078	6.623

**Table 4:** Parameters of the cantilevers.

Cantilever *i*	Length *l_i_* (μm)	Width of the Polysilicon Layer *w*_1*i*_ (μm)	Width of Metal Layer *w*_2*i*_ (μm)	Thickness of the Polysilicon Layer *h*_1_ (μm)	Thickness of the Metal Layer *h*_2_ (μm)	Initially Buckled or Unbuckled	The First Resonance Frequency *f_i_* (kHz)
Cantilever 1	150	15	5	1.5	0.5	buckled	74.38
Cantilever 2	150	15	9	1.5	0.5	buckled	68.28
Cantilever 3	200	15	5	1.5	0.5	buckled	41.72
Cantilever 4	200	15	9	1.5	0.5	buckled	38.75

**Table 5:** Results of numerical fitting for cantilevers.

Cantilever *i*	Curvature Radius *R* (μm)	Standard Deviation *σ_R_* (μm)	Abscissa of the Anchor End *x*_0_ (μm)	Standard Deviation *σ_x_* (μm)	Ordinate of the Anchor End *z*_0_ (μm)	Standard Deviation *σ_z_* (μm)
Cantilever 1	12,997.32	24.60	2.98	1.40 × 10^−^^1^	5.06 × 10^−^^3^	3.34 × 10^−^^4^
Cantilever 2	9690.63	14.89	−1.37	1.20 × 10^−^^1^	1.73 × 10^−^^3^	4.09 × 10^−^^4^
Cantilever 3	13,011.4	12.10	2.27	0.93 × 10^−^^1^	3.31 × 10^−^^3^	3.03 × 10^−^^4^
Cantilever 4	9587.3	7.33	−0.94	0.79 × 10^−^^1^	1.86 × 10^−^^3^	3.61 × 10^−^^4^

**Table 6:** Results in different sets of cantilevers.

Cantilever *i*	Young’s Modulus in the Polysilicon Layer *E*_1_ (GPa)	Young’s Modulus in the Metal Layer *E*_2_ (GPa)
Cantilever 1 and 2	156.77	68.54
Cantilever 3 and 4	146.78	82.89
Average value	151.78	75.72
Reference range reported in [24]	120~201	78

## Supplementary Materials

The following are available online at http://www.mdpi.com/2072-666X/8/7/201/s1, Figure S1: (a) A schematic of the laser Doppler vibrometer (LDV); (b) A picture of LDV system, Figure S2: (a) A schematic of the digital holographic microscopy (DHM); (b) A picture of DHM.
