# Supplementary material for: A Simple Extraction Method of Young’s Modulus for Multilayer Films in MEMS Applications
Source: Micromachines (Basel). 2017 Jun 23;8(7):201. doi: 10.3390/mi8070201 (PMC6190058; doi:10.3390/mi8070201)
Supplement: Supplementary file 1 [file micromachines-08-00201-s001.pdf]

Supplementary Materials: A Simple Extraction Method of Young's Modulus for Multilayer Films in MEMS Applications

Xin-Ge Guo, Zai-Fa Zhou, Chao Sun, Wei-Hua Li and Qing-An Huang

1. Resonance Frequency Measurement Using Laser Doppler Vibrometer (LDV) System

Laser Doppler vibrometer (LDV) (Polytech GmbH, Berlin, Germany) system is a device for non-contact vibration test on the surface. Samples under test are exposed to the laser beam from LDV (Polytech GmbH, Berlin, Germany) and the vibration will lead to the Doppler shift of the reflected laser beam from samples [1]. Thus frequencies of samples can be extracted by the Doppler shift. A schematic of it is shown in Figure S1a.

Specifically, by the beam splitter 1, the laser beam from LDV (Polytech GmbH, Berlin, Germany), whose frequency is f_0, is divided into two beams, the reference beam and the test beam. The test beam passes the beam splitter 2 and then is focused on the target surface by the lens, leading to a small facula on the target surface. A part of the laser beam, which is reflected by the target surface and then collected by LDV (Polytech GmbH, Berlin, Germany), gets through the beam splitter 3 after the reflection and scattering of beam splitter 2. After the reflection of the mirror, the reference beam, which gets through an acousto-optic modulator and the beam splitter 3, has interference with the test beam reflected [2].

Bragg Cell is adopted in the system to be the acousto-optic modulator to make the reference beam have 40M frequency shift. Output intensity, which contains the information of frequency shift, is transformed into electrical signal by the photo detector 1 and 2. After a series of circuit manipulation such as amplification, filtering and DAC, digital signal of vibration velocity can be obtained. A Polytech scanning LDV (Polytech GmbH, Berlin, Germany) system with hardware modules of MSV-400-M2 was used in the paper, as shown in Figure S1b.

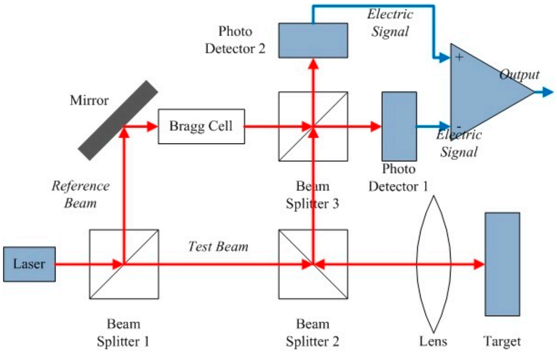

(**a**)

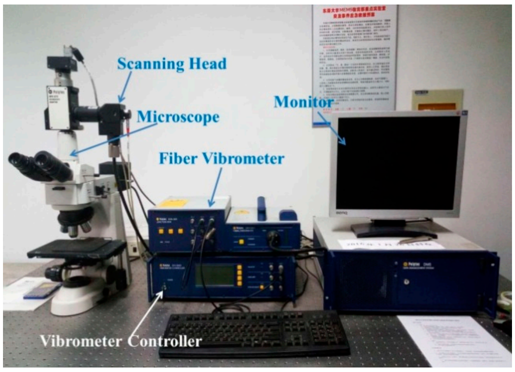

(**b**)

Figure S1. (**a**) A schematic of the laser Doppler vibrometer (LDV); (**b**) A picture of LDV system.

2. Deflection Measurement Using Digital Holographic Microscopy (DHM)

Digital holographic microscopy (DHM) (Lyncée Tech, Lausanne, Switzerland is a microscope to obtain three-dimension structure of samples using the light interferogram. It can observe samples in nanometer lever directly and instantly. And it is considered that undamaged optical measurement is achieved owing to its non-contact method.

A schematic of a reflecting DHM (Lyncée Tech, Lausanne, Switzerland is shown in Figure S2a. To have the required interferogram, the monochrome coherent light, such as laser beam, is demanded when illuminating samples. Laser beam is amplified by condenser and then is divided into two beams, the reference beam and the object beam, by beam splitter 1. Object beam gets through the microscope object by passing lens and beam splitter 2. And a facula is formed on the sample surface. Reflected by the sample surface, a part of reflected beam is collected by the DHM (Lyncée Tech, Lausanne, Switzerland and has interference with the reference beam after a series of convergence and reflection. A hologram is formed and then recorded by camera. According to the hologram, three-dimension diagram is gotten using digital reconstruction algorithm [2]. In the paper, a DHM (Lyncée Tech, Lausanne, Switzerland (R2200), which is made by Lyncée Tec SA Company, is used, as shown in Figure S2b.

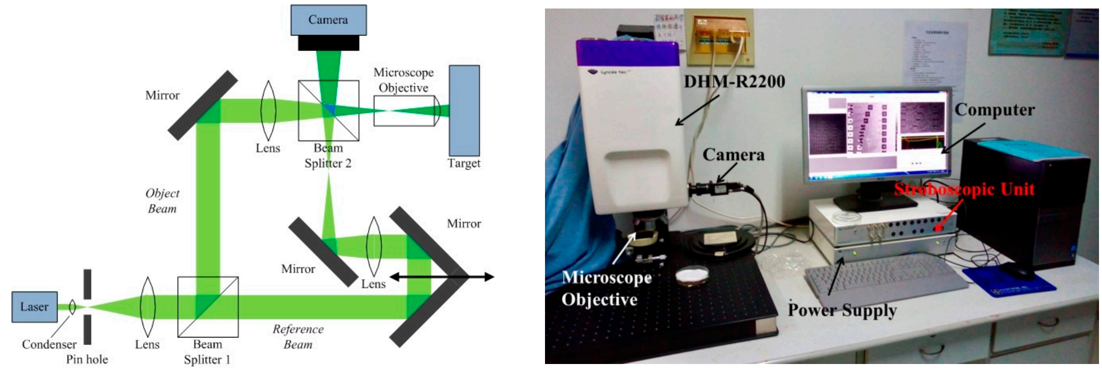

(**a**) (**b**)

Figure S2. (**a**) A schematic of the digital holographic microscopy (DHM); (**b**) A picture of DHM.

Reference

1. Ozdoganlar, O.B.; Hansche, B.D.; Carne, T.G. Experimental modal analysis for microelectromechanical systems. *Soc. Exp. Mech.* **2005**, 45, 498–506.
2. Sun, C. Study on In-Situ Extracting Method for Mechanical Properties of CMOS MEMS Multilayered Films. Ph.D. Thesis, Southeast University, Nanjing, China, November 2014. (In Chinese)
